# Curious enough to start up? How epistemic curiosity and entrepreneurial alertness influence entrepreneurship orientation and intention

**DOI:** 10.3389/fpsyg.2022.1003866

**Published:** 2022-10-14

**Authors:** Henrik Heinemann, Patrick Mussel, Philipp Schäpers

**Affiliations:** ^1^Division of Psychology of Entrepreneurship, Department of Psychology, University of Münster, Münster, North Rhine-Westphalia, Germany; ^2^Division of Personality Psychology and Psychological Assessment, Department of Education and Psychology, Freie Universität Berlin, Berlin, Germany

**Keywords:** entrepreneurship, epistemic curiosity, entrepreneurial intentions, entrepreneurial alertness, openness to experience

## Abstract

Epistemic curiosity as the desire to acquire new knowledge and ideas is considered as an important attribute for successful entrepreneurs among practitioners, yet there is lacking empirical evidence of epistemic curiosity having an effect on entrepreneurial outcomes. This study aims to put a spotlight on epistemic curiosity as a predictor for entrepreneurial intentions and orientation. We found that epistemic curiosity has a stronger influence on entrepreneurial outcomes in comparison to the Big Five personality *trait openness to experience*, which is a widely used and conceptually related predictor for entrepreneurship. Furthermore, we found evidence for a mediating role of entrepreneurial alertness which gives further insights about how personality influences the ability to recognize business opportunities and leads to the formation of entrepreneurship orientation and intentions. Our findings contribute to the field of entrepreneurship research by emphasizing that epistemic curiosity may be one of the most important personality indicators for the emergence of entrepreneurial intentions and behavior.

## Introduction

**Figure 1:**
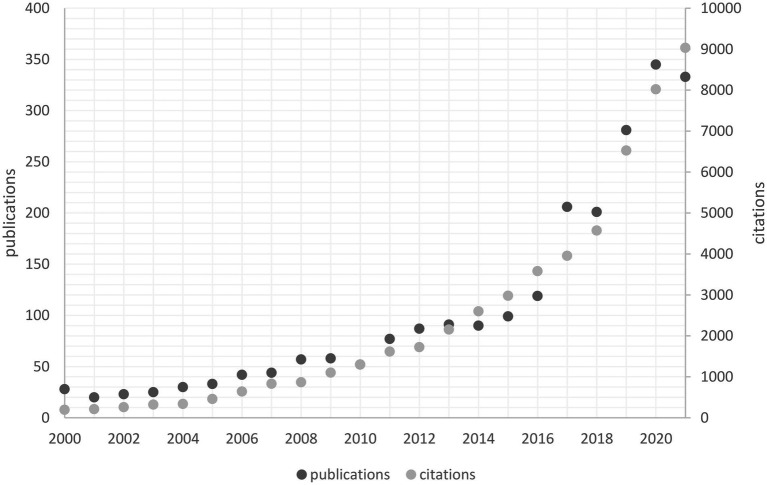
Publications and citations on Curiosity in psychology and management research from 2000 to 2021. This figure was created based on a Web of Science search for “curiosity” (25/08/2022).

Curiosity is the desire to gain new experiences and knowledge; it motivates people to learn and try something new and is a driving force for human behavior throughout many domains and stages of life (Berlyne, 1954; Loewenstein, 1994; Mussel et al., 2012; Gino, 2018; Lindholm, 2018; Litman, 2019). Interest in researching curiosity has risen in the past years across multiple fields. Thus, a Web of Science search showed that the number of citations and publications addressing curiosity has almost tripled between 2016 and 2021, with 333 publications in 2021 alone (see Figure 1). In fact, research on curiosity has found that curiosity has many positive effects on our lives, ranging from improved interpersonal relationships (Kashdan and Roberts, 2004) to social strengths (Kawamoto et al., 2017) and academic achievement (von Stumm et al., 2011). Furthermore, curiosity has long been prized in the occupational context, with many employers labeling themselves as curious, encouraging employees to be curious, and even hiring for curiosity (Mussel, 2013b). In the organizational context, curiosity has proven to be a valuable attribute for multiple work-related outcomes, including job performance, leadership, and creative performance (Harvey et al., 2009; Chang and Shih, 2019; Wagstaff et al., 2021).

Hence, curiosity seems to be a desirable attribute for finding a job in an existing organization, but does it also make someone want to start their own business? Many motivational blog entries and popular science articles point to the outstanding importance of curiosity for successfully founding a business (Goldin, 2018; Hamilton, 2019; Austin, 2020). These often highlight the dimension of curiosity that motivates people to tirelessly engage in learning new knowledge and ideas, which is conceptualized as epistemic curiosity (Berlyne, 1954). Some researchers even label it one of the “keys to entrepreneurial success” (Raine and Pandya, 2019, p. 189). In fact, researchers seem to agree that curiosity sparks innovation and creativity and influences productivity and job performance (Barron and Harrington, 1981; Reio and Wiswell, 2000; Gino, 2018). Surprisingly though, despite the apparent consensus about curiosity being one of the key attributes common to entrepreneurs, empirical evidence, and theoretical foundations about the role of curiosity in the emergence of entrepreneurship are still lacking.

We aim to contribute to entrepreneurial research by further investigating the importance of personality traits, specifically epistemic curiosity, for the emergence of entrepreneurial behavior. We contribute to the further understanding of the processes underlying the entrepreneurial personality by examining a mediational relationship between curiosity, entrepreneurial alertness, and entrepreneurial behaviors. We aim to strengthen the position of curiosity as one of the drivers of entrepreneurship tendencies, by comparing it to openness to experience as a trait strongly connected to entrepreneurial intention (Zhao and Seibert, 2006; Antoncic et al., 2015; Chan et al., 2015; Şahin et al., 2019).

Throughout this article, we will first describe the concept of epistemic curiosity and the contexts for which it is a relevant trait. In the following, we will describe the relevance of epistemic curiosity for the entrepreneurship context and compare it to openness to experience as a familiar concept which is used extensively to describe the entrepreneurial personality. Then, we will briefly describe the concept of entrepreneurial alertness and its relationship to epistemic curiosity, as we propose that curiosity is an antecedent of entrepreneurial alertness. Materials and methods are described, before we report the results from our analyses. In the last section of this article, implications for entrepreneurship researchers and practitioners are discussed.

## Literature review and hypotheses development

### Epistemic curiosity

As curiosity can be observed in many different contexts, Berlyne (1954) differentiated between a perceptual and an epistemic dimension of curiosity. Perceptual curiosity refers to the sensation of visual, auditory, and tactile stimuli, while epistemic curiosity is defined as a desire for new information that motivates one to engage in learning and exploratory behavior (Litman and Spielberger, 2003). The epistemic form of curiosity is closely related to measures of intellectual achievement and is conceptually close to familiar constructs like need for cognition and openness to ideas (Mussel, 2010). Curiosity is directly contributing to knowledge acquisition, which positively influences performance in the workplace and other contexts (Jeong and Lee, 2019; Lievens et al., 2022).

Curious behavior can be driven either by positive interest, aiming at pleasurable feelings of discovering something new, or deprivation, occurring as a response to an uncomfortable state of not-knowing (Litman, 2008). The interest (I-type) and deprivation (D-type) dimensions reflect that curiosity can be associated with both positive and negative affect, depending on whether the individual is motivated by positive anticipation or by being unsatisfied with an existing knowledge gap (Litman, 2008). Moreover, epistemic curiosity can be differentiated into specific and diverse curiosity, depending on the range of topics that are affected by an individual’s curious behavior (Mussel et al., 2012). While diverse curiosity refers to general exploration, specific curiosity is shown when people engage in trying to solve a “particular puzzle” (Hagtvedt et al., 2019, p. 1).

In general though, there seems to be a single factor underlying epistemic curiosity, which is why we conceptualize epistemic curiosity as a unitary construct in the present study (Mussel et al., 2012). Curiosity in its epistemic form has been found useful in various applied contexts and is positively related to creative performance (Hardy et al., 2017), academic learning (Litman, 2008), and work-related measures, such as job performance (Mussel, 2013b).

### Curiosity and the entrepreneurial context

What motivates people to create their own business has been a core research topic for entrepreneurship scholars in the past years (e.g., Fayolle and Liñán, 2014; Shane and Nicolaou, 2015; Fuller et al., 2018; Murnieks et al., 2020; Douglas et al., 2021). Researchers have put a lot of effort in examining which attributes and characteristics of a person are crucial for becoming a (successful) entrepreneur. They found a wide array of attributes influencing entrepreneurial behavior, ranging from personal values (e.g., Hueso et al., 2021), self-efficacy (e.g., Wilson et al., 2007), or environmental orientation (e.g., Barba-Sanchez et al., 2022), to a number of broad or specific personality traits (e.g., Zhao H. et al., 2010).

Despite the well-established use of curiosity as a predictor in other domains, its use in entrepreneurship research has been scarce. Merely one meta-analysis about the effects of career adaptability showed that curiosity can increase one’s orientation toward entrepreneurship as a positive career adaptation result (Rudolph et al., 2017). Furthermore, Syed et al. (2020) found that curiosity moderated the relationship between entrepreneurial passion and intentions and Jeraj and Antoncic (2013) were working toward a concept of context-specific entrepreneurial curiosity. These results linking curiosity to entrepreneurial outcomes are promising, but the field is lacking a solid body of research, as ample evidence cannot be found. It needs to be established whether curiosity has a substantial effect on entrepreneurial outcomes and what the nature of this effect is.

The lack of research on curiosity’s role in entrepreneurship is surprising, since being curious is highly relevant for entrepreneurs, given that entrepreneurship can be conceptualized as the identification and exploitation of business opportunities (Shane and Venkataraman, 2000). Before an opportunity can be exploited, it needs to be identified by the entrepreneur. Curiosity motivates people to ask questions, solve problems, and deal with complex theories, all of which could help them identify opportunities that have not yet been exploited by others (Litman and Spielberger, 2003).

Many great inventions and historical discoveries came about because an explorer or an inventor was curious about something and did not stop looking into it (Gino, 2018); the same attitude should help a person become a pioneer in a field of business and recognize a business opening before someone else does. Research on the recognition of entrepreneurial opportunities often focuses on the need to be alert to potential business opportunities, which enables individuals to discover or create a fit between market needs and available resources (Ardichvili et al., 2003). While this requires a certain amount of knowledge about market needs and resources, a more personality-based approach could introduce curiosity as an individual source of opportunity recognition, for multiple reasons. First, people who are driven by a desire to learn, explore, and fill knowledge gaps should be more likely to identify opportunities as a result of their exploratory behavior (Arikan et al., 2020). Curious people should also be more intrigued to act on those opportunities as they are generally driven by the desire to succeed in an environment of uncertainty (Litman and Jimerson, 2004). In this context, curiosity can work as “a catalyst for individual action,” leading individuals to engage in unknown activities such as founding a business and also to enjoy doing so (Lievens et al., 2022, p. 19). This ability to adapt and thrive in unknown settings by proactively engaging in learning and exploratory behavior is probably one of the most important benefits of curiosity for any new entrepreneur. Second, curiosity activates behaviors that could directly lead to entrepreneurial actions, such as identifying a promising business idea. In a business context, the pressing urge to fill an encountered knowledge gap will lead the curious to be highly invested in their market research, possibly leading them to recognize a problem or issue with an existing market supply. Problem identification can be considered an initial stage of founding a start-up and is, therefore, the first entrepreneurial behavior that curiosity has a direct impact on (Frese and Gielnik, 2014). Third, as an epistemically curious individual, an entrepreneur will be highly motivated to solve relevant intellectual problems and come up with creative solutions that might turn into a business opportunity (Barron and Harrington, 1981; Baggen et al., 2015). As a consequence, more curious people should be better at identifying business opportunities.

Curious individuals enjoy contexts of uncertainty and novelty more than others and are better equipped to cope in those unknown and complex situations (Mussel, 2013b). The interest dimension of curiosity is especially associated with optimistic self-regulatory strategies, perseverance, and accepting higher risks in connection with exploration (Lievens et al., 2022). For an entrepreneur, these might be critical skills, e.g., when it comes to creating a business plan, preparing a pitch, or “going the extra-mile.” Because of these direct links between curious behavior and entrepreneurial behavior, a curious individual should be more likely to think about starting their own business.

We expect that curiosity influences people’s orientation and attitudes toward entrepreneurial topics and that more curious people have higher entrepreneurial intentions, which we define as a person’s intent to start their own business and be self-employed (Krueger, 1993). Thus, we propose that curiosity is related to entrepreneurial outcomes, especially in advance of recognizing business opportunities. According to Ajzen (1985), intention is one of the best predictors for behavior, which is why we assess entrepreneurial intention as a determinant of entrepreneurial behavior.

*Hypothesis 1*: Epistemic curiosity is positively related to entrepreneurship outcomes (i.e., entrepreneurial intentions and individual entrepreneurial orientation).

### Openness to experience and epistemic curiosity

While curiosity has been widely unrecognized by entrepreneurship research, broad personality domains like the Big Five personality factors have been at the center of many efforts to predict entrepreneurial outcomes (Zhao and Seibert, 2006; Zhao H. et al., 2010; Brandstätter, 2011; Frese and Gielnik, 2014; Antoncic et al., 2015). Along with *conscientiousness* and *neuroticism* (negatively correlated), *openness to experience* has shown a significant association with starting a new business (Frese and Gielnik, 2014). Nevertheless, there is reasonable concern that broad personality traits might not be optimal for predicting entrepreneurial outcomes (Postigo et al., 2021). Rauch and Frese (2007) argued that the predicting traits should be matched to entrepreneurial tasks and showed in a meta-analysis that narrow personality traits, like innovativeness, proactive personality, and risk propensity, were better predictors for entrepreneurship than broad personality dimensions (Leutner et al., 2014). According to the symmetry principle (Wittmann, 1988), the predictive validity of a construct suffers when it contains criterion-irrelevant components or the predictor has a different level of generality than the criterion (Schulze et al., 2021). As such, this means that broad personality domains (e.g., openness to experience or conscientiousness) are too far up in the hierarchical order of personality traits to effectively predict a relatively narrow criterion like entrepreneurship. Thus, one should refer to narrower traits in the hierarchy, like the specific Big Five sub-facets or other lower-order traits (Schulze et al., 2021). As an example, studies measuring conscientiousness include both the facets achievement motive and dependability. These two facets are specific traits, but only one of them (achievement motive) is strongly correlated with the criterion (business success), whereas the other (dependability) is not (Zhao and Seibert, 2006). As a result, the overall score for conscientiousness’ correlation with business success is lower than for its facet achievement motive, which drew Rauch and Frese (2007) to conclude that the specific personality trait of achievement motive is a better predictor for entrepreneurship than conscientiousness. Even though complex measures are a comfortable choice, a growing research body argues that selecting specific measures for both sides of the prediction equation can improve the understanding of the relationship at hand (Tett et al., 2003).

Further, any construct used to predict entrepreneurial outcomes should directly “match personality with work characteristics” (Rauch and Frese, 2007, p. 358). Consequently, this means that in our study, predictors should not include components that are unmatched with entrepreneurial work characteristics; following a confirmatory research strategy by making sure all of our predicting traits are related to the tasks that occur for the work of an entrepreneur (Tett and Christiansen, 2007).

Analogous to the example for conscientiousness mentioned above, the factor openness to experience in its entirety seems too broad and heterogeneous for our purpose (Mussel et al., 2011), even though it is commonly used to predict entrepreneurial criteria like intentions or business creation (Zhao and Seibert, 2006; Awwad and Al-Aseer, 2021). As it also contains sub-facets that are irrelevant for entrepreneurial outcomes (e.g., openness to feelings and openness to esthetics; Mussel et al., 2011) we should reconsider whether openness to experience fits with the criterion of predicting entrepreneurial criteria. Considering this, epistemic curiosity offers a more specific personality trait from the openness to experience spectrum but that is free from irrelevant sub-facets. Searching for more specific personality traits in this context, the next step would be to examine the six sub-facets of this Big Five factor. While some of the sub-facets (e.g., openness to esthetics and feelings) clearly seem to be irrelevant for entrepreneurship, others are a better match for entrepreneurial tasks. Next to adventurousness and liberalism, especially openness to ideas (also referred to as intellect; Goldberg et al., 2006) seems a good fit for this cause, as it drives intellectual exploration and coming up with new ideas (Mussel, 2013a).

Openness to experiences shows certain similarities with epistemic curiosity; in fact, it is hard to establish discriminant validity between the two concepts. Curiosity is a more agentic trait and explains behavior more directly (Harrison and Dossinger, 2017). In contrast, the openness to experience facets are passive traits and do not hold any motivational aspects (Harrison and Dossinger, 2017). On a sub-dimensional level, deprivation-type epistemic curiosity shows associations with conscientiousness, as it refers to the perseverant acquisition of knowledge rather than wide exploration and imagination (Litman and Mussel, 2013). Furthermore, in an attempt to establish construct validity for epistemic curiosity, Mussel (2013a) showed that work-related curiosity best predicts vocational interest in occupations from the entrepreneurial context.

In line with the demands issued by Rauch and Frese (2007)—that good predictors for entrepreneurship need to be narrow traits matched to entrepreneurial tasks—epistemic curiosity may present a predictor on the right level of the hierarchy of personality traits matched to entrepreneurial tasks and free from task-irrelevant sub-facets. Convergent validity with factors closely related to entrepreneurial outcomes (i.e., the conscientiousness facet achievement, creativity, and intellectual stimulation), strengthens the assumption of curiosity being relevant for entrepreneurship (Litman and Spielberger, 2003; Mussel, 2010; Hardy et al., 2017). Additionally, epistemic curiosity had incremental validity over openness to experience in predicting work-related criteria like job performance (Mussel, 2013b), which is why we hypothesize that curiosity will also show incremental validity in predicting various measures of entrepreneurship.

*Hypothesis 2*: Epistemic curiosity predicts entrepreneurship (i.e., entrepreneurial intentions and individual entrepreneurial orientation) beyond the effect of openness to experience.

### Entrepreneurial alertness

In past research, the successful recognition of business opportunities has often been explained using the concept of entrepreneurial alertness (Neneh, 2019; Sharma, 2019; Chavoushi et al., 2021; Tang et al., 2021; Lanivich et al., 2022). Entrepreneurial alertness is an individual’s ability to identify business opportunities, which are not recognized by others (Kirzner, 1983). The concept aims to explain how entrepreneurs identify new opportunities to start a business, with the assumption that entrepreneurs are generally more alert to opportunities (Gaglio and Katz, 2001; Tang et al., 2012). Kirzner (1997) as the first one to use the term entrepreneurial alertness, argued that business opportunities are not necessarily *created* by particular entrepreneurs, in contrast they need to be *found* after they emerge due to suboptimal market processes that result in a market imbalance (Sharma, 2019). While alertness is widely accepted to be an antecedent for entrepreneurial behavior, there are different theories about the theoretical foundations, with some arguing for behavioral explanations (Kaish and Gilad, 1991) and others highlighting cognitive capacities, especially creativity and general mental ability (Gaglio and Katz, 2001; Baron and Ensley, 2006). In original conceptualization of Kirzner (1997), he laid great importance on the availability of information that leads to the recognition of profit opportunities. As some people had better access to information than others, it was easier for them to recognize opportunities without searching for them (see Sharma, 2019). However, the conceptualization of alertness has evolved in its definition since Kirzner, leading to multiple streams of research about the theoretical foundations of the concept, such as cognitive abilities, social networks, and personality traits, as Lanivich et al. (2022) point out in a systematic review on alertness (also see Sharma, 2019).

### Epistemic curiosity and entrepreneurial alertness

Following a personality-based approach on entrepreneurial alertness, alertness, and the recognition of possible business opportunities might be influenced by the personality trait curiosity. As a “critical first step” of the entrepreneurial process, recognizing business opportunities might depend on an entrepreneur’s exploratory and learning behavior (Ardichvili et al., 2003; Chavoushi et al., 2021, p.2). Being alert to new possibilities does not require possessing information but rather the ability and motivation to acquire new information, which should make learning and exploratory behavior important factors for alertness (Uy et al., 2015). People who can “think outside the box” and are proactively trying to acquire new information should be more alert to new possibilities and should, effectively, be more successful at identifying new business opportunities (Hu et al., 2018). We propose that epistemic curiosity contributes to individual differences in people’s alertness to business opportunities, specifically that curious people are more entrepreneurially alert.

*Hypothesis 3*: Epistemic curiosity is positively related to entrepreneurial alertness.

Drawing from an unclear state of research about the underlying foundations of individual alertness (Frese and Gielnik, 2014; Lee Lim et al., 2014; Lanivich et al., 2022), we propose that curiosity is not just an antecedent of alertness but is actually the driving force behind entrepreneurial alertness’ effect on entrepreneurial outcomes. Curious behavior, like learning new skills, adapting to changing environments, trying to solve complex problems, or simply reading the news on a newly discovered topic, helps people to be aware of evolving opportunities and improves their entrepreneurial alertness. People who engage in learning and exploratory behavior and are motivated by the desire to fill knowledge gaps should score higher on both entrepreneurial alertness and entrepreneurship measures.

In this manner, we propose that the positive effect curiosity has on entrepreneurship is transmitted through entrepreneurial alertness. We formulate our theorizing on the conceptual link between curiosity and alertness as both refer to the active search for ideas and information that can lead to the discovery of business opportunities, with curiosity as a specific personality trait and alertness as a cognitive skill (Tang et al., 2012; Lievens et al., 2022). Various research has shown that entrepreneurial alertness substantially contributes to the formation of entrepreneurial intentions (McMullen and Shepherd, 2006; Hu et al., 2018). In turn, alertness itself is strongly connected to personality and is positively influenced by different underlying competencies (Obschonka et al., 2017). Assessing the relationship between personality and entrepreneurial intention, personality traits like creativity, boundaryless mindset, and proactivity affected intentions toward entrepreneurship *via* mediating processes involving entrepreneurial alertness (Lee Lim et al., 2014; Uy et al., 2015; Obschonka et al., 2017; Hu et al., 2018). Thus, we argue that curiosity manifests itself onto recognizing business opportunities, for which alertness is a crucial skill and we propose a mediational model, stating that curiosity leads to entrepreneurial alertness, which effectively leads to entrepreneurial behavior. Hence, we posit:

*Hypothesis 4*: Entrepreneurial alertness mediates the relationship between epistemic curiosity and entrepreneurship (i.e., entrepreneurial intentions and individual entrepreneurial orientation).

## Materials and methods

### Sample

An *a priori* power analysis computed with g^*^power 3.1.9.7 resulted in a sample size of 295 participants required to test our hypotheses with a statistical power of 1−*β* = 0.95, on a significance level of α = 0.05 (Faul et al., 2009). Specifically, we assumed an effect of Cohen’s *f^2^* = 0.044, responding to an assumed increase of Δ*R^2^ =* 0.04 in a hierarchical linear regression with three predictors. The assumed effect size corresponds to findings about the incremental validity of curiosity above openness to experience for the prediction of job performance (Mussel, 2013b).

**Table 1:** Descriptive sample characteristics.

	**Entrepreneur** **(*N* = 115)**	**Non-Entrepreneur** **(*N* = 181)**	**Overall** **(*N* = 296)**
**Gender**			
Female	71 (61.7%)	135 (74.6%)	206 (69.6%)
Male	35 (30.4%)	38 (21.0%)	73 (24.7%)
Other/ No answer	9 (7.9%)	8 (4.4%)	17 (5.7%)
**Age**			
Mean (SD)	39.0 (13.1)	26.1 (9.41)	31.1 (12.7)
**Education**			
Master’s or higher	26 (22.6%)	9 (5.1%)	35 (11.1%)
Bachelor’s	37 (32.2%)	52 (28.7%)	89 (30.1%)
Associate Degree	12 (10.4%)	13 (7.2%)	25 (8.4%)
Trade/technical/vocational training	6 (5.2%)	1 (0.6%)	7 (2.4%)
Some college credit, no degree	22 (19.1%)	55 (30.4%)	77 (26.0%)
(Some) High school	12 (10.5%)	51 (28.2%)	63 (21.3%)

*N* = 296. Participants were on average 31.1 years old (*SD* = 12.7), and participant age ranged from 18 to 74 years.

Of 383 participants, 356 people finished the survey and were compensated for their participation with a monetary reward (7.05% drop-out rate). A total of 60 participants were excluded from the analyses due to peculiar responses to quality-monitoring items (for further details, see section careless responding).^1^ The final sample consisted of 296 participants from 18 to 74 years old, residing in the United States (Table 1). The participants were 31.12 years old on average (*SD* = 12.67 years) and 69.59% were female. In this sample, 16 participants did not identify as male or female, and one participant chose the option “prefer not to answer.” Most participants were in an employed working position at the time of the survey (58.45%). Of the total sample, 38.85% (115) reported that they had started a business in the past or were currently self-employed.

**Table 2:** Technical specifications of the study.

Population	Entrepreneurs in the United States: 31 million (GEM (Global Entrepreneurship Monitor), 2022)
Sampling technique	Online panel prolific.co Screening criteria: US American citizen, first language English 50%: self-described entrepreneur
Method of data collection	Online survey
Sample size	296
Dates of data collection	11/07/2021–26/07/2021
Pre-Registration	https://aspredicted.org/GMR_W5N
Data available	https://osf.io/95vbq/?view_only=48fe194b2deb441bb61c2be56b5485b7

Data collection was anonymous; no data were saved on the servers of prolific.co.

The majority of participants (50.34%) had a university degree (associate degree or higher), while 48.31% held a high school degree, and 1.35% did not finish high school.

### Procedure

We designed an online survey using the experimental software platform Unipark (EFS Survey, Questback GmbH). We recruited a sample of American adults *via* the online research platform Prolific,^2^^,^^3^ which offers a diverse participant population using crowdsourcing for behavioral research (Peer et al., 2017). We used screening functions of the platform to specifically reach entrepreneurs, which we defined as people who are currently running their own business or have done so in the past (Table 2). Before data collection started, the study design and hypotheses were pre-registered on aspredicted.org.^4^ Participants were asked to complete a test battery consisting of multiple questionnaires to assess the variables depicted below. Participation was voluntary and anonymous, the participants had both the chance to cancel their participation at any time or self-exclude their data from further processing after finishing the study. The participants provided their written informed consent to participate in this study. The mean duration for completing the survey was 14 min, after completion they were automatically referred to the panel website.

### Independent variables

The independent variables were measured by established scales that have been used previously in multiple studies (Heaven and Bucci, 2001; Lee-Ross, 2017; Hu et al., 2018) and have shown sufficient reliability and validity.

#### Epistemic curiosity

To assess epistemic curiosity, we used the English version of the Work-Related Curiosity Scale (WORCS; Mussel et al., 2012). The WORCS consists of 10 items, e.g., “I am interested in how my contribution impacts the company,” that are rated on a Likert scale ranging from 1 (*does not apply at all*) to 7 (*fully applies*). This scale offers a work context-specific assessment, which we chose because our study focuses on the work-related impact that curiosity might have. The reliability of the scale was good, with α = 0.87. As an alternative measure for epistemic curiosity, we used the two-dimensional 10-item I/D EC-Scale, which includes five items each to measure the interest-type and deprivation-type dimensions of epistemic curiosity on a context-unspecific level (Litman et al., 2010). This scale produces a more differentiated view on epistemic curiosity at the facet level, allowing us to explore whether the two dimensions impact entrepreneurship differently. Participants were asked to rate their feelings toward each item on a four-point Likert scale from 1 (*almost never*) to 4 (*almost always*; Litman et al., 2010). Reliabilities for the scales ranged from α = 0.85 to α = 0.87.

#### Openness to experience

To measure the personality trait openness to experience according to Costa and McCrae (2008), we used a 60-item scale from the International Personality Item Pool (IPIP; Goldberg et al., 2006) with 10 items for each of six facets, namely imagination, artistic interests, emotionality, adventurousness, intellect, and liberalism, measured on a five-point Likert scale. Sample items are “I have a vivid imagination” (imagination) and “I like to solve complex problems” (intellect). The facets are similar to those of the commonly used NEO-PI-R by Costa and McCrae (2008) but are labeled differently; for example, the intellect facet can be interpreted analogously to the openness to ideas facet from the NEO-PI-R. Reliability of the openness to experience scale was α = 0.91. The highest internal consistency was found for the facet intellect (α = 0.86); the facets with the lowest reliabilities were adventurousness and artistic interest (α = 0.80).

#### Entrepreneurial alertness

Entrepreneurial alertness was measured using the 13-item scale by Tang et al. (2012). Example items included “I have a gut feeling about potential opportunities” and “I always keep an eye out for new business ideas when looking for information,” rated on seven-point Likert scales from 1 (*strongly disagree*) to 7 (*strongly agree*). The instrument contains three separate scales, namely (1) scanning and search, (2) association and connection, and (3) evaluation and judgment. Reliabilities (α) of the three scales ranged from 0.77 to 0.89.

#### Affinity for technology

Additionally, the battery included the Affinity for Technology Interaction scale to detect possible moderating effects (Franke et al., 2019). For this scale, we used a six-point Likert scale ranging from 1 (*completely disagree*) to 6 (*completely agree*) to rate the nine items (Franke et al., 2019). The reliability of the scale was good, with α = 0.87.

#### Careless responding

Finally, we assessed careless responding to identify and exclude irregular responders. Meade and Craig (2012) recommended including bogus items which are used to detect careless responding (Anderson et al., 1984; Levashina et al., 2009): Participants were asked about their familiarity with made-up techniques (e.g., “I am trained at using Johnson’s dyadic approach of avoiding conflict in work teams”; see Levashina et al., 2009). In addition, we inserted two instructed response items (e.g., “To monitor quality, please respond with a two for this item,” see Meade and Craig, 2012). Participants were excluded if they failed to insert the correct response for more than one instructed response item or if they indicated to agree with at least one bogus item.

### Dependent variables

Entrepreneurial outcomes were assessed using two separate variables from the entrepreneurial context that are central outcomes of entrepreneurship, namely entrepreneurial intentions and individual entrepreneurial orientation (Baron and Ensley, 2006; Zhao H. et al., 2010; Bolton and Lane, 2012).^5^

#### Entrepreneurial intentions

Entrepreneurial intentions were measured using the Entrepreneurial Intention Questionnaire (Liñán and Chen, 2009), which contains six items measured on a seven-point Likert scale. A sample item is “I am determined to create a company in the future.” The reliability of the questionnaire was good, with α = 0.96. As we also included entrepreneurs in the sample, we added the question “Have you ever started your own business?” to later explore differences between entrepreneurs and non-entrepreneurs. Participants were additionally asked to estimate the probability that they will start their own business in the next 5 years on a scale from 1 to 100 percent (Krueger et al., 2000). This was added as a purely exploratory assessment to gain a more direct estimation of how manifested the participant’s thought of starting a business was when considering a fixed time frame.

#### Entrepreneurial orientation

As a second measure for entrepreneurship, we assessed participant’s individual entrepreneurial orientation (IEO), using 10 items from Bolton and Lane (2012). The measure includes items for the dimensions risk taking, innovativeness, and proactive personality rated on a five-point Likert scale ranging from 1 (*strongly disagree*) to 5 (*strongly agree*). The reliability of the IEO scale was sufficient, with α = 0.81.

### Analysis strategy

Group differences between entrepreneurs and non-entrepreneurs were explored using a one-way MANOVA. We performed a linear regression analysis to identify the possible influence that epistemic curiosity has on entrepreneurial intentions and individual entrepreneurial orientation. Multiple regression analyses were used to examine the amount of variance in entrepreneurship that could be explained by epistemic curiosity compared to openness to experience in a model using both as predictors for each entrepreneurship outcome variable. Values for openness to experience were computed by taking the mean value over all openness facets of a participant. Analogously, the mean of all three alertness facets formed a person’s entrepreneurial alertness score. All hypotheses were tested at factor level. Mediation analysis was used following recommendations by Zhao X. et al. (2010) to investigate a mediational relationship between epistemic curiosity, entrepreneurial alertness, and entrepreneurship. The indirect effect was tested for significance using a bias-corrected bootstrapping procedure (MacKinnon et al., 2004). Data and Supplementary materials are available at https://osf.io/95vbq/?view_only=48fe194b2deb441bb61c2be56b5485b7.

## Results

### Preliminary analyses

Before we tested our hypotheses, preliminary analyses were conducted to describe the data, explore group differences, consider the reliabilities of the scales, and check for common method variance. We tested group differences between entrepreneurs and non-entrepreneurs to explore whether they already differed in our dependent and independent variables before looking closer at the relationship between those variables.

**Figure 2:**
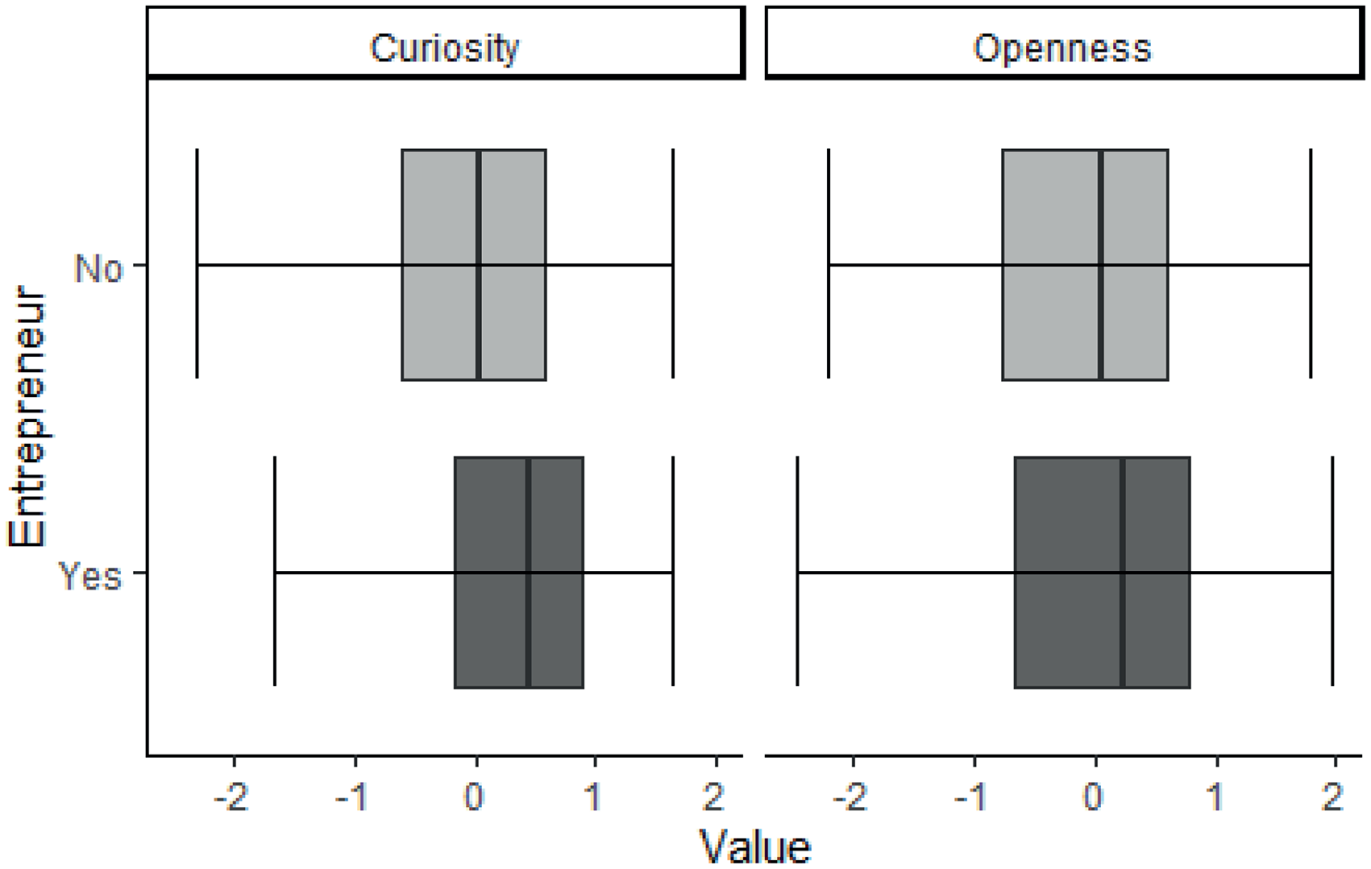
Values of curiosity and openness for entrepreneurs and non-entrepreneurs. Boxplots mark the median for each group and interquartile range for the standardized values of openness to experience and epistemic curiosity, respectively. Non-entrepreneurs in light gray, entrepreneurs in dark gray.

We first explored whether entrepreneurs and non-entrepreneurs differed in curiosity and openness to experience, as finding differences for only one of the variables might already indicate a disparate importance for becoming an entrepreneur, before even running more fine-grained analyses when testing for the hypotheses. To test for group differences between entrepreneurs and non-entrepreneurs, we performed a one-way MANOVA with the main predictors and criteria for the upcoming hypotheses tests. Results of the MANOVA can be seen in Table 3, showing a significant multivariate effect of entrepreneurial status [Pillai’s trace = 0.29; *F*(2, 291) = 29.40; *p* < 0.001]. Univariate comparisons found group differences for all entrepreneurial outcomes and epistemic curiosity, but not for openness to experience (see Table 3), the alpha level was Bonferroni-corrected (0.05/4 = 0.0125) as we tested for four separate dependent variables. These differences are illustrated in Figure 2, showing significant differences in epistemic curiosity values [η_p_^2^ = 0.04; *F*(1, 294) = 13.54; *p* < 0.001], whereas the groups showed similar median values for openness to experience [η_p_^2^ < 0.01; *F*(1, 294) = 1.69; *p =* 0.195]. Figure 2 shows that entrepreneurs reported higher levels of epistemic curiosity than non-entrepreneurs, yet no significant differences were found for openness to experience. Entrepreneurial intention correlated with both I-type (*r = 0*.30) and D-type epistemic curiosity (*r = 0*.28), yet the size of the correlations was not significantly different (*p = 0*.75; 95% KI*r_I-type_*-*r_D-type_* = [−0.11;0.16]).

**Table 3:** One-way MANOVA results for group differences between entrepreneurs and non-entrepreneurs.

	Value	*F*	**Hypotheses *df***	**Error *df***	*p*			
Pillai’s trace	0.29	29.40	4	291	< 0.001			
Wilk’s lambda	0.71	29.40	4	291	< 0.001			
Hotelling’s trace	0.40	29.40	4	291	< 0.001			
	**Entrepreneurs (*n* = 115)**		**Non-Entrepreneurs (*n* = 181)**			**Hypotheses *df***	**Error *df***	***p***
	***M***	***SD***	***M***	***SD***	***F***			
Predictors								
Epistemic Curiosity Openness to	5.72	0.88	5.32	0.94	13.54	1	294	< 0.001
Experience	3.93	0.42	3.87	0.38	1.69	1	294	0.195
Criteria								
Entrepreneurial Intention	5.06	1.55	2.96	1.67	118.07	1	294	< 0.001
Individual Entrepreneurial Orientation	3.79	0.65	3.44	0.62	21.24	1	294	< 0.001

*M* and *SD* are used to represent mean and standard deviation, respectively. *F* statistics and degrees of freedom are reported.

Participants who reported that they had already started their own business at some point rated the probability that they will start another one at 60%, on average.

Age and gender had an effect on entrepreneurial outcomes but not on epistemic curiosity. The level of education was not related to entrepreneurial outcomes.^6^ Descriptive statistics, Pearson correlations, and reliabilities for the independent and dependent variables can be seen in Table 4 (reliabilities on the diagonal).

**Table 4:** Means, standard deviations, and correlations.

Variable	*M*	*SD*	1	2	3	4	5	6	7	8	9
											
1. Openness to Experience	3.89	0.40									
2. Epistemic Curiosity	5.47	0.94	0.41^**^								
3. I-type Curiosity	3.15	0.64	0.59^**^	0.60^**^							
4. D-type Curiosity	2.37	0.71	0.18^**^	0.43^**^	0.34^**^						
5. E. Alertness	4.94	0.88	0.33^**^	0.57^**^	0.47^**^	0.43^**^					
6. Scan & Search	5.09	1.03	0.34^**^	0.56^**^	0.49^**^	0.35^**^	0.80^**^				
7. Association & Connection	5.17	1.13	0.37^**^	0.47^**^	0.45^**^	0.43^**^	0.81^**^	0.52^**^			
8. Evaluation & Judgment	4.56	1.16	0.10	0.33^**^	0.20^**^	0.25^**^	0.77^**^	0.42^**^	0.39^**^		
9. E. Intentions	3.78	1.92	0.15^**^	0.39^**^	0.30^**^	0.28^**^	0.49^**^	0.51^**^	0.37^**^	0.31^**^	
10. IEO	3.58	0.65	0.33^**^	0.50^**^	0.37^**^	0.39^**^	0.65^**^	0.54^**^	0.49^**^	0.52^**^	0.43^**^

*M* and *SD* are used to represent mean and standard deviation, respectively.

* indicates *p* < 0.05;

** indicates *p* < 0.01.

As for all studies using self-report data, it was necessary to check whether our data were biased by common method variance (CMV). If a method factor accounts for a major amount of variance in the variables, it can distort item validities and the covariation between latent variables (MacKenzie and Podsakoff, 2012). To check for CMV, we applied Harman’s one-factor test to see whether a single factor accounted for much of the covariance in our variables in an exploratory factor analysis (Podsakoff et al., 2003). This method is commonly used for identifying potential common method bias (Fuller et al., 2016). Following recommendations by Podsakoff et al. (2003), all items were loaded into an exploratory factor analysis; the result showed that the proportion of variance explained by a single general factor was 41% (*RMSEA* = 0.164; 95%CI [0.143,0.187]), therefore not exceeding the critical 50% level. Even though the test does not control for method variance, it is unlikely that common method bias is a contaminant in our study, as the test indicated the presence of two separate factors, and therefore did not detect problematic variance explained by a single factor. In a confirmatory factors analysis, the single-factor model did not show a good fit with the data (CFI = 0.538) in comparison to the proposed model ( Δ χ^2^ = 1663.98; *p* < 0.001).

### Hypotheses testing

Hypothesis 1 proposed that epistemic curiosity is positively related to the entrepreneurial outcomes of entrepreneurial intention and individual entrepreneurial orientation. Hypothesis 1 was supported, as epistemic curiosity showed significant correlations with entrepreneurial intention (*r = 0*.39; *p* < 0.01) and individual entrepreneurial orientation (*r = 0*.50; *p* < 0.01). A linear regression resulted in a significant regression weight of 0.80 (*SE =* 0.11; *p < 0*.017) for curiosity predicting entrepreneurial intentions and individual entrepreneurial orientation (*β* = 0.34; *SE =* 0.04; *p < 0*.017). The *α* level was corrected to adjust for alpha cumulation (α/number of tests = 0.05/3 = 0.017), as we repeatedly tested for three^7^ dependent variables addressing the same hypothesis (Cabin and Mitchell, 2000).

To test if epistemic curiosity predicts entrepreneurship outcomes beyond what is explained by openness to experience (Hypothesis 2), we conducted an ordinary least squares regression analysis comparing a model using openness to experience and epistemic curiosity as predictors for entrepreneurship to a model using just openness to experience as a predictor. Results for the regression analyses can be seen in Table 5. The requirements for linear regression were met, as the data showed a linear relationship between predictors and criteria, and multivariate normality could also be observed when plotting the data. Furthermore, we found multicollinearity not to be a problem in our analysis, as the variance inflation factor (*VIF* = 1.20) was below 10, which is considered uncritical according to common rules (O’Brien, 2007). The assumption of homoscedasticity could also be kept, as a studentized Breusch–Pagan test (Breusch and Pagan, 1979) showed a non-significant result (*p* = 0.059).

**Table 5:** Regression results for Hypothesis 2.

Predictor	Entrepreneurial intention					Individual entrepreneurial orientation				
	*beta*	*beta* 95% CI [LL, UL]	*r*	Fit *R^2^*	Difference Δ*R^2^*	*beta*	*beta* 95% CI [LL, UL]	*r*	Fit *R^2^*	Difference Δ*R^2^*
Model 1										
Openness to Experience	0.15^**^	[0.04, 0.27]	0.15^**^			0.33^**^	[0.22, 0.43]	0.33^**^		
				0.023^**^					0.106^**^	
				95% CI [0.00,0.07]					95% CI [0.05,0.17]	
Model 2										
Openness to Experience	−0.01	[−0.12, 0.11]	0.15^**^			0.15^**^	[0.04, 0.25]	0.33^**^		
										
Epistemic Curiosity	0.39^**^	[0.28, 0.51]	0.39^**^			0.44^**^	[0.33, 0.54]	0.50^**^		
				0.152^**^	0.128^**^				0.263^**^	0.158^**^
				95% CI [0.08,0.22]	95% CI [0.06, 0.20]				95% CI [0.18,0.34]	95% CI [0.09, 0.23]
										

Beta indicates the standardized regression weights. *r* represents the zero-order correlation. LL and UL indicate the lower and upper limits of a confidence interval, respectively. Δ*R*^2^ represents the difference between coefficients of determination of model 1 and 2.

** indicates *p* < 0.01.

Adding epistemic curiosity to the model as a second predictor led to a 12.8% increase of variance explained in entrepreneurial intentions ( $\Delta R^{2}$ = 0.128; *p* < 0.01). Table 5 also depicts the results for a regression using the same arrangement of predictors but using individual entrepreneurial orientation as the criterion. Epistemic curiosity accounted for an increase in $R^{2}$ of $\Delta R^{2}$ = 0.16 (*p* < 0.01) when added last to the regression, confirming Hypothesis 2.

Hypothesis 3 stated that epistemic curiosity is positively related to entrepreneurial alertness. Linear regression results supported this assumption and showed a positive influence of curiosity on alertness (*β* = 0.57; *p* < 0.001); regression results can be seen in Table 6. Thus, Hypothesis 3 was supported.

**Table 6:** Regression results using entrepreneurial alertness as the criterion.

Predictor	*b*	*beta*	*beta* 95% CI [LL, UL]	*r*	Fit
(Intercept)	2.02^**^				
Epistemic Curiosity	0.53^**^	0.57	[0.48, 0.66]	0.57^**^	
					*R^2^* = 0.324^**^
					95%CI [0.24,0.40]
					

A significant b weight indicates the beta weight is also significant. *b* represents unstandardized regression weights. Beta indicates the standardized regression weights. *r* represents the zero-order correlation.

** indicates *p* < 0.01.

**Figure 3:**
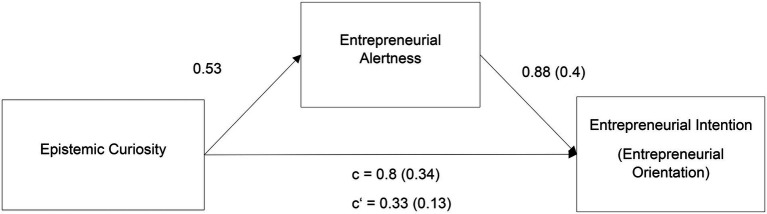
Mediational model for the prediction of entrepreneurial intention and orientation. Coefficients written on the paths are direct effects. “c” is the effect of epistemic curiosity on the criterion with the effect of alertness included. Values in brackets are direct effects on the criterion entrepreneurial orientation, analogously.

Next, we proposed a mediational model of entrepreneurial alertness mediating the relationship between curiosity and entrepreneurship (Hypothesis 4). Figure 3 shows the mediational model, in which the total and direct effects of curiosity and alertness on entrepreneurial intentions and individual entrepreneurial orientation are illustrated. The established mediation can be classified as a complementary mediation, as both a direct and an indirect effect exists, both of which positively influence entrepreneurial intention (Zhao X. et al., 2010). Still, entrepreneurial alertness only partly mediates the relationship, as the direct effect of epistemic curiosity was not reduced to zero when controlling for the mediator (Baron and Kenny, 1986). To illustrate the intermediary effect, a bootstrapping procedure with 10,000 iterations was performed to test for significance of the indirect effect. The results can be seen in Table 7. The indirect effect is 0.47 for curiosity on entrepreneurial intention, with a 95% bootstrap CI of 0.31 and 0.66. The total and direct effects of the mediational model can be seen in Figure 3. The same steps were also performed on a model using individual entrepreneurial orientation as the criterion; here, a significant mediation effect was also found when individual entrepreneurial orientation was used as the entrepreneurship criterion. For this bootstrapping regression, which was performed with the same number of iterations, the indirect effect was 0.22 (*p* < 0.01; 95% CI[0.15, 0.29]). Thus, Hypothesis 4 was also supported.

**Table 7:** Results for mediation analysis from epistemic curiosity to entrepreneurial alertness to entrepreneurial intentions.

Variables	Estimate	*SE*	*t*	*df*	*p*
Epistemic Curiosity → Entrepreneurial Intentions					
Direct effect	0.33^**^	0.13	2.64	293	< 0.01
Total effect	0.8^**^	0.11	7.25	294	< 0.01
**Variables**	**Estimate**	**Boot SE**	**Boot LLCI**	**Boot ULCI**	***p***
Epistemic Curiosity → Entrepreneurial Alertness → Entrepreneurial Intentions Indirect effect	0.47^**^	0.09	0.31	0.66	< 0.01

*N* = 296. Confidence intervals are set at 95% from the bootstrap analysis with 10,000 bootstrap resamples. SE, standard error; LLCI, lower level of confidence interval; and ULCI, upper level of confidence interval.

** indicates *p* < 0.01.

### Additional analyses

In addition to the analyses required for testing the hypotheses, we ran additional analyses with the six sub-facets of openness to experience in comparison to epistemic curiosity’s effect on entrepreneurial outcomes. We examined a reversed mediational model using curiosity as the mediator to evaluate the fit of our proposed mediational model. Furthermore, we ran exploratory analyses using participants’ technology affinity as a possible moderator.

#### Openness to experience sub-facets

In a multiple regression analysis using all openness to experiences sub-facets as predictors for entrepreneurial intention, only intellect (*β = 0*.66; *p < 0.*001) and adventurousness (*β = 0*.60; *p < 0*.01) showed significant positive regression weights. Analogously, intellect (*β = 0*.32; *p < 0.*001) and adventurousness (*β = 0*.31; *p < 0.*001) were the only significant positive regression weights predicting entrepreneurial orientation. Next, we tested whether curiosity significantly increased the proportion of explained variance over these two sub-facets in the prediction of entrepreneurial outcomes. In a linear regression, intellect explained 8.2% of the variance in entrepreneurial intention, yet the amount of variance explained almost doubled when epistemic curiosity was added as a second predictor (Δ $R^{2}$ = 0.078; *p* < 0.01). Similar results were found for the analogous regression analysis performed with individual entrepreneurial orientation as the criterion.

The adventurousness sub-facet also accounted for a significant part of the variance in participants’ entrepreneurial intentions ( $R^{2}$ = 0.053; *p* < 0.01) but less so than intellect. When epistemic curiosity was added to the model, it again greatly increased the amount of variance explained (Δ $R^{2}$ = 0.110; *p* < 0.01). Similarly, adventurousness also contributed to the determination coefficient in entrepreneurial orientation ( $R^{2}$ = 0.177; *p* < 0.01), whereas epistemic curiosity to this model added another 10% in explained variance (Δ $R^{2}$ = 0.101; *p* < 0.01).

#### Reversed mediation model

To evaluate the fit of our proposed mediational model, we compared it to a mediational model exchanging the mediator and the predictor. When using epistemic curiosity as a mediator for the relationship between alertness and entrepreneurial intention, no significant indirect effect was detectable using a Sobel Test (*p* = 0.214). This indicates that the order of effects that are proposed in our mediational model according to our theorizing are reasonable, as the order of the independent and moderating variables was not trivial.

#### Affinity for technology

For exploratory purposes, we investigated whether the direction or strength of curiosity’s effect on entrepreneurship depended on participants’ expression of an affinity for interacting with technology. An increasing percentage of newly founded start-ups have a digital and highly technologized character (Wu and Atkinson, 2017). Thus, because technology is important for the start-up sector, we were interested in whether our data showed an interplay between a participant’s affinity for technology and curiosity. For example, people highly engaged in technology might either refrain from starting a business, as they are aware of the strength of the market competition in the technology sector, or they might be even more inclined to start their own tech business given their strong technological knowledge.

After performing a moderated regression analysis for this purpose, the data did not significantly show that technology affinity had a moderating effect on the relationship between curiosity and entrepreneurial intention (*β* = 0.10; *p* = 0.385) or entrepreneurial orientation (*β* = 0.04; *p* = 0.236). Apart from that, affinity for technology itself was positively associated with entrepreneurial intention (*β* = 0.35; *p* < 0.1) and orientation (*β* = 0.10; *p* < 0.5). The results from the moderated regression analysis are depicted in Table 8.

**Table 8:** Moderated regression results using entrepreneurial intentions as the criterion.

Predictor	*b*	*SE*	*t*	*p*	95% CI	Fit
					[LL, UL]	
(Intercept)	3.75^**^	0.11	35.17	< 0.01	[3.54, 3.96]	
Epistemic Curiosity	0.70^**^	0.12	5.96	< 0.01	[0.47, 0.93]	
ATI	0.35^**^	0.11	3.10	< 0.01	[0.13, 0.57]	
Epistemic Curiosity × ATI	0.10	0.11	0.87	0.39	[−0.12, 0.32]	
						*R^2^* = 0.182^**^
						95% CI[0.10,0.25]

ATI indicates affinity for technology index. *b* represents unstandardized regression weights. SE, standard error; LL and UL indicate the lower and upper limits of a confidence interval, respectively.

** indicates *p* < 0.01.

## Discussion

With the present study, we intended to contribute to entrepreneurship research by emphasizing the importance of curiosity for the emergence of entrepreneurial intention and orientation. We found that curiosity is of particular importance for recognizing business opportunities, which is an important step in the entrepreneurial journey (Chavoushi et al., 2021). To outline curiosity’s importance in this process, we proposed that entrepreneurial alertness is positively influenced by curiosity and impacts entrepreneurial outcomes. Specifically, we found evidence of alertness mediating the positive relationship between curiosity and entrepreneurial intention and orientation.

Even though openness to experience is often used to predict entrepreneurial tendencies, we only found weak correlations between this factor and entrepreneurial outcomes; the effect sizes were in line with meta-analytic results (Zhao and Seibert, 2006). We proposed epistemic curiosity as a conceptually better-suited predictor for entrepreneurship. Our data showed that epistemic curiosity predicts entrepreneurship above the effect of openness to experience. This indicates that curiosity can explain parts of variance in entrepreneurial outcomes that exceed what can be explained by openness to experience.

Furthermore, we proposed a mediational model, which was supported by the data, showing that entrepreneurial alertness mediates the relationship between curiosity and entrepreneurship. In contrast to prior result that found the effect of creativity and proactive personality on entrepreneurship to be completely mediated by alertness (Hu et al., 2018); the effect of curiosity was only partly mediated. This means that curiosity has an impact on entrepreneurship apart from what is transported *via* alertness, indicating that there are more mechanisms and processes affected by curiosity that lead to entrepreneurship; these need to be further investigated. The present results contribute to entrepreneurship research by strengthening the claims about the importance and nature of curiosity’s role for the emergence of individual entrepreneurship (Harrison and Dossinger, 2017; Rudolph et al., 2017; Syed et al., 2020; Lievens et al., 2022).

### Theoretical implications

The size of the effects observed in the regression analyses show that curiosity is not only related to entrepreneurship but seems to be among the strongest predictors for an individual’s tendency to start a business. In the magnitude of the correlations that were found, curiosity even exceeded the effects of self-efficacy, autonomy, and risk propensity, which were the strongest predictors of entrepreneurial outcomes in meta-analytic examinations (Rauch and Frese, 2007; Zhao H. et al., 2010). Putting these results into perspective, our study delivers first and initial evidence for considering epistemic curiosity as one of the most important traits of the entrepreneurial personality.

Analyzing group differences between entrepreneurs and non-entrepreneurs, a noteworthy finding of this study is that they significantly differed in their expression of curiosity, yet no significant differences were found for openness to experience. This yields important theoretical contributions, since we found openness to predict the intentions and orientation toward starting a business, yet actually running a business seemed less related to openness to experience. There is a considerable gap between intention and behavior in the entrepreneurial context (Harima et al., 2021). It might be an interesting avenue for future research to determine if actions are more likely to follow entrepreneurial intentions if the person is more curious. Curiosity as an action-oriented trait could moderate the intention-behavior relationship, as the missing link that Kautonen et al. (2015, p. 670) described as “any personality attribute that refers to a preference for doing versus thinking, for example a preference for learning by doing and experimenting.”

Finding differences in the values of openness and epistemic curiosity between entrepreneurs and non-entrepreneurs also points toward further evidence that curiosity and familiar constructs can be clearly distinguished. The present study shows that curiosity explains parts of variance in entrepreneurial outcomes that exceed what can be explained by openness to experience. As a consequence, the critical behaviors that lead to the formation of entrepreneurial intentions do not seem to be a result of the underlying mechanisms that curiosity and openness share but especially of those in which they differ. In contrast to openness to experience and its facets, curiosity is more active, agentic, and motivating (Harrison and Dossinger, 2017). Especially curiosity as a feeling of deprivation drives individuals to invest high levels of energy to acquire new information (Lievens et al., 2022). This initiative and active orientation toward new information in an uncertain environment seems to be necessary to make the decision to become an entrepreneur. In contrast, being passively *open* to the idea of founding a business might not be sufficient to form entrepreneurial tendencies or recognize business opportunities, as the results of this study suggest. The differences between the constructs are visible in the results as, epistemic curiosity showed incremental validity not only above the effect of openness to experience but also above the sub-facets of the Big Five factor.

Despite conceptual differences between interest-and deprivation-type curiosity, both were equally important for the entrepreneurial outcomes, as their effects were not significantly different. They impacted the outcomes equally, yet they may influence entrepreneurship from different approaches, thus fostering different behavioral expressions (Litman, 2008; Lievens et al., 2022); such expressions could not be observed in this study, as we only assessed the outcomes and not the actions preceding them.

The mediation of curiosity’s effect on entrepreneurship *via* alertness implies that certain behaviors are activated by curiosity, whereby a curious mind leads to entrepreneurial behavior that has not yet been identified. The strong role of entrepreneurial alertness hints that this gap might be filled by actions that allow an individual to recognize entrepreneurial opportunities. Actively approaching new information in the context of exploration seems likely to be the key quality that empowers curious individuals to engage in entrepreneurial activities.

We contribute to entrepreneurial alertness research, as we provide further evidence that entrepreneurial alertness works as a strong predictor for entrepreneurship, building on prior theory that personality traits like creativity and proactivity influence a prospective entrepreneurs ability to recognize opportunities, which in turn leads to higher entrepreneurial intentions (Lee Lim et al., 2014; Uy et al., 2015). It seems that a commonality of many different predictors for entrepreneurship is that their influence is at least partly transported *via* one construct. This yields the following question: What explains this special position that entrepreneurial alertness seems to hold? Some argue that the concept of alertness itself is problematic, as it does not have an *a priori* meaning; alertness can only be observed once a person has actually identified an opportunity (McMullen and Shepherd, 2006). Therefore, critics argue that entrepreneurial alertness cannot be used as a “universal attribute of entrepreneurial individuals independent of the system in which they operate” (McMullen and Shepherd, 2006, p. 144). This would make alertness less of a predictor for entrepreneurial behavior but a kind of entrepreneurial behavior itself. In this context, it seems to be at least just as interesting to find out more about the antecedents of entrepreneurial alertness, which requires an integrated approach because alertness cannot be explained using exclusively behavioral or cognitive constructs (Frese and Gielnik, 2014). The present study contributes to this line of research, as epistemic curiosity can be added to the range of antecedents of alertness with a noteworthy impact.

The relevance of curiosity for entrepreneurship should not go unnoticed when examining conceptual models for the development of entrepreneurial behavior, such as the model proposed by Frese and Gielnik (2014). How exactly curiosity’s strong influence is conveyed in the processes of developing an orientation toward entrepreneurial action should be subject to future research. Integrating epistemic curiosity in a more domain-specific reference has been the focus of a series of publications by Jeraj and Antoncic (2013), who introduced the concept of entrepreneurial curiosity, because they regarded other types of curiosity as too broad to be applied in an entrepreneurial context. They developed a measure specifically for the entrepreneurial context, referring to entrepreneurial tasks like market research, company improvement, and marketing strategies, which they report to be independent of other types of curiosity and linked to a range of constructs close to entrepreneurship, e.g., innovativeness and opportunity creation (Jeraj, 2014; Arikan et al., 2020). We encourage the domain-specific application of curiosity, yet the present results show that epistemic curiosity is already well suited to predict entrepreneurial outcomes. Whereas an entrepreneurial curiosity measure (Jeraj and Marič, 2013) can be used for established entrepreneurs that already had experience with entrepreneurial tasks, measures of epistemic curiosity have the advantage of being applicable to persons who have no prior connection with entrepreneurship.

### Practical implications

As we found that epistemic curiosity is a promising predictor for entrepreneurship, the construct can be utilized in multiple practical appliances in the start-up context. There is a growing market for start-up academies and start-up coaches that aim to support nascent entrepreneurs by providing individual training and assistance (Hofmann, 2021). To construct appropriate coaching plans and further develop the clients’ entrepreneurial qualities, psychological assessments can be helpful in identifying an individual’s needs and opportunities for improvement (e.g., Entrepreneurial Mindset Profile; Davis et al., 2016). Notably, these measures may be enriched by adding epistemic curiosity as a construct, possibly leading to more accurate assessments and predictions of entrepreneurial potential. It might even be preferred over previously used measures, for example those that include openness to experience. Epistemic curiosity should, therefore, play an essential role in the conception of new instruments for measuring what is often called the *entrepreneurial mindset*. In another avenue, epistemic curiosity may also be important to investors, as they may want to factor in an entrepreneur’s curiosity when deciding whether to invest their start-up. Another important domain where epistemic curiosity can contribute is the promotion of nascent entrepreneurs. Considerable effort has been undertaken by governments to increase the rate of innovations and to support their country’s start-up sector by funding institutions that aim to support entrepreneurs on their journey with advice, training, and financial support (Zinke et al., 2018). Research on the entrepreneurial psychology is relevant for the success of institutions fostering nascent entrepreneurs and should have a direct effect on the strategic alignment of these institutions (Barba-Sanchez et al., 2022). As a predictor for entrepreneurial behavior, curiosity can contribute to the work of these institutions, for example by identifying the need for further training. Showing young people that starting their own business is a promising career path—especially for highly curious people—should be used to encourage young adults and students to follow through on their ideas. In the context of entrepreneurial education initiatives, assessing and fostering curiosity in students could help increase the rate of young entrepreneurs and spark innovations (Zappe et al., 2018).

### Limitations and future research directions

Further research efforts should investigate our findings using a wider array of methods. To achieve more profound knowledge about whether curiosity can also contribute to explain the success of an entrepreneur’s business throughout later stages of the start-up process, future investigations should also use longitudinal designs. Future research efforts could go beyond early entrepreneurial outcomes like entrepreneurial intention and orientation and focus on criteria further along the entrepreneurial journey. These should include more extensive criteria to measure the success of a business, for example economic measures like financial status and growth rate, but also well-being and job-satisfaction of entrepreneurs and their employees.

A limitation concerning the comparability of the different curiosity measures refers to possible frame-of-reference effects (Schmit et al., 1995). The outcomes and the work-related curiosity scale both referred to an occupational context, whereas the second curiosity measure and the openness to experience scale did not refer to a specific context, which might explain the higher correlations between the work-related curiosity measure and the outcomes (Schulze et al., 2021). As the unspecific curiosity measure also strongly correlated with the entrepreneurial outcomes, the frame-of-reference effect seems at least uncritical.

A next step starting from the present results might be to explore exactly what kinds of curious behavior lead an entrepreneur to close the gap between recognizing an opportunity and making the decision to actually start a business. In this context, developing a conceptual model might be appropriate to connect behavioral expressions rooted in epistemic curiosity, such as active information seeking and knowledge acquisition, with entrepreneurial activities that can be objectively observed. Even though we did not find a moderating effect of participants’ technology affinity, it is still likely that curiosity is of different importance for entrepreneurs in different sectors. In this context, future research should investigate how specific curiosity that is focused on a single field of interest (Hagtvedt et al., 2019), for example new technologies, can foster the process of engaging in an entrepreneurial activity. A differentiated view on individuals’ interests and curiosity concerning specific domains could lead to different results for the relationship between curiosity and entrepreneurial outcomes.

## Conclusion

Our study contributes to the entrepreneurship research by examining a key personality trait of the entrepreneurial personality and encouraging further research on this relationship. Empirical analyses from a quantitative study show that epistemic curiosity is closely related to entrepreneurial intention and orientation. In this context, epistemic curiosity was observed to be a better predictor for entrepreneurship than the broad Big-Five-factor openness to experiences and any sub-facets of openness to experiences. The size of the effect was in line with some of the most important predictors for business creation, such as self-efficacy and autonomy (Frese and Gielnik, 2014). Examining the nature of the relationship between curiosity and entrepreneurial outcomes, we found that the effect was mediated by entrepreneurial alertness. With this, we offer further evidence for personality traits activating the ability to identify opportunities which effectively leads to the formation of entrepreneurial intention and orientation toward an entrepreneurial career (Ardichvili et al., 2003; Shane and Nicolaou, 2015). We outline the role of alertness in this process, which builds on previous research establishing alertness as a mediator for multiple antecedents of entrepreneurial behavior (Lee Lim et al., 2014; Uy et al., 2015; Hu et al., 2018). Taking a closer look at this relationship would be interesting for future research efforts. Specifically, we are curious about how epistemic curiosity can be integrated in existing entrepreneurial models (e.g., Ardichvili and Cardozo, 2000; Douglas et al., 2021) and how valuable it will prove to be for predicting business success and other criteria in the entrepreneurial context.

## Data availability statement

The datasets presented in this study are made available in the Open Science Framework (https://osf.io/) and can be found at: https://osf.io/95vbq/?view_only=48fe194b2deb441bb61c2be56b5485b7.

## Ethics statement

Ethical review and approval was not required for the study on human participants in accordance with the local legislation and institutional requirements. The participants provided their written informed consent to participate in this study.

## Author contributions

HH was responsible for drafting the manuscript and implemented the survey and conducted statistical analyses. HH, PM, and PS played important roles in the conceptualization process and in the writing of the manuscript. All authors contributed to the article and approved the submitted version.

## Funding

HH and PS would like to thank the State of North Rhine-Westphalia’s Ministry of Economic Affairs, Industry, Climate Action and Energy as well as the Exzellenz Start-up Center. NRW program at the REACH – EUREGIO Start-Up Center for their kind support of our work.

## Conflict of interest

The authors declare that the research was conducted in the absence of any commercial or financial relationships that could be construed as a potential conflict of interest.

## Publisher’s note

All claims expressed in this article are solely those of the authors and do not necessarily represent those of their affiliated organizations, or those of the publisher, the editors and the reviewers. Any product that may be evaluated in this article, or claim that may be made by its manufacturer, is not guaranteed or endorsed by the publisher.

## Footnotes

^1^The results of our analyses did not substantially change when the 60 people were included in the analyses. Compensation did not depend on the answers to the quality-monitoring items.

^2^ www.prolific.co

^3^Prolific and other online crowdsourcing platforms have shown to be reliable and valid tools for behavioral research that can successfully replicate experimental studies from laboratory settings (Crump et al., 2013; Palan and Schitter, 2018).

^4^ https://aspredicted.org/GMR_W5N

^5^Recognition of business opportunities was assessed as a third measure for entrepreneurial behavior. Results are available in Appendix A (see Electronic Supplementary material).

^6^Age was positively related to entrepreneurial outcomes. Men showed higher entrepreneurial intention and orientation than women. Results for the preliminary analyses are available in the Supplementary material.

^7^We also assessed the number of business opportunities identified in the past 5 years as a third measure of entrepreneurial performance. To improve readability, the results of the opportunity determination are not provided. Due to its ordinal-scale nature, ordinal logistic regression analyses were performed. Notably, results are in line with the other two dependent variables, confirming all hypotheses. More details about these analyses can be requested from the first author of this study and can be found in the Supplementary material.
