# Supplementary material for: Curious enough to start up? How epistemic curiosity and entrepreneurial alertness influence entrepreneurship orientation and intention
Source: Front Psychol. 2022 Oct 14;13:1003866. doi: 10.3389/fpsyg.2022.1003866 (PMC9614078; doi:10.3389/fpsyg.2022.1003866)
Supplement: Supplementary file 1 [file Data_Sheet_1.docx]

**Electronic Supplementary Material**

**Appendix A**

**Validity** As some of our variables have shown to be somewhat similar in recent studies, we examined the construct validity of our predicting variables to rule out that our predictors are essentially measuring the same construct, before starting to test the hypotheses (Lievens et al., 2022). Convergent validity was established between two different measures of epistemic curiosity, as a two-dimensional and a unidimensional measure for curiosity were used (*r*= 0.60 with I-type; *r*= 0.43 with D-type curiosity). The unidimensional WORCS measure (Mussel et al., 2012) was used as a general one-factor measure for epistemic curiosity to test the hypotheses (see next chapter), whereas the two-dimensional measure was added to allow more nuanced analyses, aiming to explore if interest- and deprivation-type curiosity impact entrepreneurial outcomes differently. In line with recent findings by Mussel (2010), discriminant validity could not be manifested between curiosity and openness to experiences (*r =*.41) and openness to ideas (*r =* .54), as they showed significant positive associations. Differences between the interest and deprivation type indicate that curiosity as a feeling of deprivation is conceptually more distanced from the openness to experiences spectrum than interest-type curiosity which was strongly associated with openness to experiences (*r* = .59) and intellect (*r* = .65).

**Preliminary Analyses Details**

A Wilcoxon rank-sum test confirmed that entrepreneurs also identified more business opportunities than non-entrepreneurs (*W =*16184;*p*< .001). Also, participants who reported that they had already started their own business at some point, rated the probability that they will start another one at 60% average. In contrast, non-entrepreneurs reported an average chance of 29% that they would someday start a business themselves. Men showed higher entrepreneurial intentions (*M*= 4.43; *SD* = 1.81) than women (*M* = 3.55; *SD* = 1.92; *t*(277) = -3.50, *p <* .001), yet no significant gender differences were found for epistemic curiosity (*F*(3, 292) = 0.40; *p*= .752). Men on average rated the probability that they will start their own business at 48%, whereas women on average reported a probability of 40%. Age was positively related to entrepreneurship (e.g., *r =*.34 with entrepreneurial intention). Significant group differences were also found for individual entrepreneurial orientation and opportunity identification. Men scored higher on entrepreneurial orientation (*M* = 3.73; *SD* = 0.68) compared to women (*M*= 3.52; *SD* = 0.63; *t*(277) = -2.29, *p =*.024). Mann-Whitney-Wilcoxon rank-sum test showed that men also scored higher in identifying business opportunities (*M* = 3.14; *SD* = 2.05) than women (*M* = 2.24; *SD* = 1.68; *W* = 5430, *p =*<.001). The level of education did not have an effect on the entrepreneurial outcomes in a MANOVA performed for exploratory purposes (Pillai’s trace = 0.11; *F*(24, 861) = 1.31; *p*= .147).

**Opportunity Identification Hypothesis Testing**

The third measure for entrepreneurship that we assessed, asked people for the number of opportunities they had identified in the past five years and how many of them they actively pursued. 124 participants responded that they did not identify any business opportunity in the past five years, 58 participants reported one identified opportunity, 48 two opportunities, 28 three, and about ten people each reported four, five, six to ten and more than ten opportunities. Because only a few people were found in the higher order categories, we categorized the originally eight categories into four. Starting with zero opportunities (124 participants), second category is “one or two” (106 participants), then “three to five” (47 participants) and finally “six or more opportunities” (19 participants). Due to the nature of the variable opportunity identification, ordinal logistic regression analyses were computed to explore the relationship between the independent variables and opportunity identification.

Addressing hypothesis 1, the ordered logit regression coefficient for epistemic curiosity was significant (*β =*.60, *SE =*0.13; *p*< .01), indicating that epistemic curiosity has a positive influence on the number of opportunities identified. The odds ratio indicated a positive relationship of a nearly two fold increase in log odds in opportunity identification (*OR =*1.83; 95% CI[1.43, 2.38]), for every one unit increase of epistemic curiosity. The same procedure was performed for the number of opportunities that were pursued by the participant. The proportional odds logistic regression resulted in a significant positive influence of epistemic curiosity (*β =*.83, *SE =*0.16; *p*< .01). The odds ratio for this model was *OR* = 2.02, meaning that an increase of one unit in epistemic curiosity increases the odds of pursuing an opportunity by the factor 2.02. Concerning hypothesis 2, we ran ordinal logistic regressions adding openness to experiences and intellect as predictors. To compare which model is a better fit for the data, we performed likelihood ratio tests. The test statistic was not significant, indicating that the less complex model using just curiosity was a better fit for the data than a model additionally including either openness to experiences (*LR* (291) = 0.01; *p =*0.91) or the intellect facet (*LR* (291) = 2.14; *p =*0.14). The analyses performed with opportunity identification as the dependent variable show similar results as for the other outcomes, indicating that curiosity influences the identification of business opportunities in a similar way as it influences entrepreneurial intention and orientation.
